# Supplementary material for: Colloidal transport by light induced gradients of active pressure
Source: Nat Commun. 2023 Jul 13;14:4191. doi: 10.1038/s41467-023-39974-5 (PMC10344923; doi:10.1038/s41467-023-39974-5)
Supplement: Supplementary file 1 — Supplementary Information [file 41467_2023_39974_MOESM1_ESM.pdf]

Supplementary material of: Colloidal transport by light induced gradients of active pressure

Nicola Pellicciotta[1,2*], Matteo Paoluzzi[3], Dario Buonomo[1], Giacomo Frangipane[1,2], Luca Angelani[4,1] and Roberto Di Leonardo[1,2*]

[1]Dipartimento di Fisica, Sapienza Università di Roma, Piazzale A. Moro 5, Roma, 00185, Italy.
[2]NANOTEC-CNR, Soft and Living Matter Laboratory, Institute of Nanotechnology, Piazzale A. Moro 5, Roma, 00185, Italy.
[3]Departament de Física de la Matèria Condensada, Universitat de Barcelona, C. Martí Franquès 1, Barcelona, 08028,Spain.
[4]ISC-CNR, Institute for Complex Systems, Piazzale A. Moro 5, Roma, 00185, Italy.

*Corresponding author(s). E-mail(s): npellicciotta@gmail.com; roberto.dileonardo@uniroma1.it;
Contributing authors: matteopaoluzzi@ub.edu ; buonomo.1554431@studenti.uniroma1.it; giacomo.frangipane@uniroma1.it; luca.angelani@roma1.infn.it;

Supplementary Notes

1 - Measurement of bacteria density. To vary cell density in the sample, we took advantage of the steady decrease of swimming bacteria in the field of view over time. This kinetic process arises because the bacteria surrounding the illuminated area are not swimming. Then, the number of bacteria going outside the illuminated area is not balanced, leading to a continuous draining of swimming bacteria from the camera field of view. Thus, taking consecutive measurements of the same particles at different time points allowed us to quantify the propulsion at different bacterial densities. To extract bacteria density in the sample, we assume cell density is proportional to the acquired dark field image. Knowing the cell concentration at the beginning of the experiment, we can extract the density of the cell at any given time during the experiment. We assume that bacterial density is proportional to dark field image $\rho_d(r)$, modulated by a spatially varying envelope due to heterogeneity in illumination, plus a background from the experimental chamber and cell buffer. We accounted for the light heterogeneity by acquiring 100 frames at 25 fps at the beginning of the experiments when the bacterial density is homogeneous, and the optical density of bacteria is known (OD_0). These frames are firstly temporally averaged and then spatially filtered with a Gaussian kernel with a large standard deviation (300 pixels). We denote this image with ρ_0. The background corrections are measured by acquiring frames of the sample chamber filled only with motility buffer and then temporally averaged, ρ_{bg}. Finally, the bacterial density, expressed in optical density, can be found using the following expression:

$$\rho(r) = \mathrm{OD}_0(\rho_d(r) - \rho_{bg})/(\rho_0 - \rho_{bg}).$$

2 - Numerical Model. As a numerical model, we consider molecular dynamics simulations of elongated run-and-tumble particles in two spatial dimensions. The system is composed of N swimmers labelled by $i = 1, ..., N$ [1–3]. Indicating with $\mathbf{v}_i$ the translational velocity of the swimmer i and with $\boldsymbol{\omega}_i$ the rotational velocity, the time evolution of the system (in the overdamped regime) is given by

$$\mathbf{v}_i = \mathbf{M}_i \cdot \mathbf{F}_i \tag{1}$$

$$\boldsymbol{\omega}_i = \mathbf{K}_i \cdot \mathbf{T}_i \tag{2}$$

where we have introduced the translational and rotational mobility matrices

$$\mathbf{M}_i = m_{\|}\hat{\mathbf{e}}_i \otimes \hat{\mathbf{e}}_i + m_{\perp} (\mathbf{1} - \hat{\mathbf{e}}_i \otimes \hat{\mathbf{e}}_i) \tag{3}$$

$$\mathbf{K}_i = k_{\perp} (\mathbf{1} - \hat{\mathbf{e}}_i \otimes \hat{\mathbf{e}}_i) \ , \tag{4}$$

with $\hat{\mathbf{e}}_i$ the swimming direction, $\otimes$ indicates the dyadic product ($\mathbf{1}$ is the identity matrix). Each swimmer is represented by a chain of p disks of diameter $\ell/2$ aligned along $\hat{\mathbf{e}}_i$. We indicate with $\mathbf{r}_i$ the center of mass of the ith swimmer. The position $\mathbf{r}_i^{\beta}$, with $\beta = 1, ..., p$, of the β-th disk composing the i-th swimmer is given by

$$\mathbf{r}_i^{\beta} = \mathbf{r}_i + \delta\mathbf{r}_i^{\beta} \ . \tag{5}$$

Here we consider elongated swimmers with $p = 2$ so that $\delta\mathbf{r}_i^1 = -\hat{\mathbf{e}}_i\ell/4$ and $\delta\mathbf{r}_i^2 = +\hat{\mathbf{e}}_i\ell/4$ (circular swimmers correspond to $p = 1$). We consider only repulsive interaction due to a pair potential r^{-12}.

The total force $\mathbf{F}_i$ and the total torque $\mathbf{T}_i$ on the i-th swimmer are

$$\mathbf{F}_i = f_0(\mathbf{r}_i)\hat{\mathbf{e}}_i(1 - \sigma_i) + \sum_{j \neq i, \alpha, \beta} \mathbf{f}(\mathbf{r}_i^{\alpha} - \mathbf{r}_j^{\beta}) + \sum_{\alpha} \mathbf{f}_{bead}(\mathbf{r}_i^{\alpha}) \tag{6}$$

$$\mathbf{T}_i = \mathbf{t}_r^i \sigma_i + \sum_{j \neq i, \alpha, \beta} \delta\mathbf{r}_i^{\alpha} \times \mathbf{f}(\mathbf{r}_i^{\alpha} - \mathbf{r}_j^{\beta}) + \sum_{\alpha} \delta\mathbf{r}_i^{\alpha} \times \mathbf{f}_{bead}(\mathbf{r}_i^{\alpha}) \, . \tag{7}$$

We have introduced a state variable σ_i, with value 0 in the running state and 1 in the tumbling state. During the running state each swimmer is self-propelled along $\hat{\mathbf{e}}_i$ with self-propulsion speed $v_i^s = m_{\|} f_0(\mathbf{r}_i)$. The self-propulsion force $f_0(\mathbf{r}_i)$ is updated according to the light pattern so that the self-propulsion speed v_i^s depend on the position of the swimmers. In the tumbling state, the random torque $\mathbf{t}_r^i$ rotates the swimming direction $\hat{\mathbf{e}}_i$ at the tumbling rate, α. To mimic the behavior of *E. coli* bacteria, the tumbling state has a finite lifetime given by $\alpha^{-1}/10$. The force between bead and swimmer is given by $\sum_{\alpha} \mathbf{f}_{bead}(\mathbf{r}_i^{\alpha})$. The equation of motion for the colloidal bead of radius a is

$$\mathbf{v}_b = \mu_b \mathbf{F}_b \tag{8}$$

with $\mathbf{v}_b$ indicating the velocity of the colloid, μ_b the mobility, and $\mathbf{F}_b$ the mechanical force resulting from the interaction with the swimmers.

We choose units such that $\ell = m_{\parallel} = 1$ and use $\alpha = 0.1$, $k_{\perp} = 4.8$ and $m_{\perp} = 0.87$. Eqs. (1) have been solved numerically using a second-order Runge-Kutta scheme with a time-step $\Delta t = 10^{-3}$ for $N_t = 10^6$ steps. We have considered system composed of $N \in [3084, 3192]$ swimmers in a box of side $L \in [48, 200]$ (with periodic boundary conditions) so that the packing fraction ϕ varies within $\phi \in [0.03, 0.53]$.

In the experiments, the cell area packing fraction ϕ can be estimated from the cell concentration (measured in optical density units) and considering that most of the bacteria accumulate at the bottom and top surfaces (persistence length greater than the sample cell height) [4]. Using our cell growth protocol, we have that the cell concentration is $N_c = 10^9$ cells/ml for an optical density OD=1 [5], and cells with an average length $l = 2.6 \pm 0.6\mu$m and diameter $\sigma = 0.86 \pm 0.07\mu$m. Assuming that half of the bacteria in the sample accumulate at the bottom surface, the number of cells on a surface of 1μm^2 area is $N_b = N_c h/2$, where h=20 μm is the chamber height. Then, the area packing fraction ϕ is the number of cells at the bottom times the cross-sectional area of a bacterium, $\phi = N_b l \sigma \approx 0.02$ for OD =1. We found that the best quantitative agreement between simulations and experiments is reached when we consider $\phi = 0.028$ for OD=1, which is not too far from the estimated value considering the large deviations in the cell size.

We have considered a bead of size $a = 5\ell$ and the radius of the light template is $R = 8a$. The mobility of the colloid is set to $\mu_b = 0.1$. We set the self-propulsion velocity $v_i^s = 1$ outside the light template and $v_i^s = 1/2$ inside the template. We have also considered the case with a background velocity $v_i^s = v_0$ and two light patterns that individuate regions $v_i^s = v_{+/-}$. In this case, we set $v_0 = 1$ and we vary $v_{+/-} \in [0, 1]$.

Swim force density. The swim force density is defined as $\mathbf{f}_S(\mathbf{r}) = \gamma v(\mathbf{r})\mathbf{m}(\mathbf{r})$, with $\mathbf{m}(\mathbf{r})$ the polarization field, $\gamma = 1/m_{\parallel}$ the viscous drag along the self-propulsion direction and $v(\mathbf{r})$ the speed pattern imposed with light. In the numerical simulations the polarization field was calculated using the formula:

$$\mathbf{m}(x, y) = \frac{1}{\Delta^2} \sum_i B(x_i - x, y_i - y, \Delta)\, \hat{\mathbf{e}}_i \tag{9}$$

with $(x_i, y_i) = \mathbf{r}_i$ and $\hat{\mathbf{e}}_i$ the position and self-propulsion direction of the ith cell and $B(x, y, \Delta)$ the box function:

$$B(x, y, \Delta) = \begin{cases} 1 \text{ if } |x_i - x| < \Delta \text{ and } |y_i - y| < \Delta \\ 0 \text{ otherwise} \end{cases} \tag{10}$$

and Δ the size of the box defining the spatial resolution of the field ($\Delta = 1.5$ in simulation units). The polarization is then averaged over time.

Direct pressure. The direct, interaction pressure term is calculated using the usual virial formula for 2D systems:

$$p_D(x,y) = \frac{1}{4\Delta^2} \sum_{i,\alpha} \sum_{j\neq i,\beta} B(x_i^\alpha - x, y_i^\alpha - y, \Delta) B(x_j^\beta - x, y_j^\beta - y, \Delta + \delta) f(r_{ij}^{\alpha\beta}) r_{ij}^{\alpha\beta}$$

with $r_{ij}^{\alpha\beta} = \|\mathbf{r}_i^\alpha - \mathbf{r}_j^\beta\|$

Supplementary figures

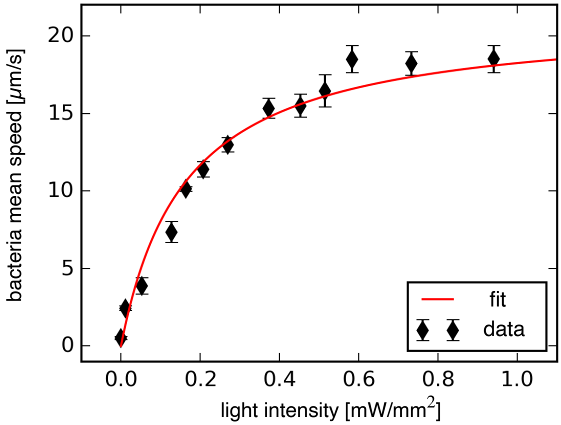

Supplementary Figure 1

Bacteria speed can be regulated by green light intensity. The effect of light on the speed distribution of our strain was quantified by monitoring bacterial dynamics with Differential Dynamic Microscopy (DDM) [6]. We projected a chessboard pattern consisting of 12 different levels of light intensity. Each light intensity level was randomly assigned to a chessboard area to avoid systematic light intensity spatial gradients. Successively, we acquired high-speed videos (1000 frames, 100 fps), and we measured the speed distributions on each illuminated area with DDM) [6, 7]. This procedure was repeated 12 times after randomly reassigning the positions of the light intensities in the same field of view, and the speed distributions are averaged over these repetitions. The reported speeds are an average of over three fields of view. Data are well represented by the following model $y = (v_M)/(1+I/(k+I))+C$ [7] (fit red line), where I is the light intensity and v_M,k,C are fit parameters. In our case we found $v_M = 21 \pm 1$μm/s, $k = 0.16 \pm 0.03$mW/mm^2 and $C = 0 \pm 0.8$μm/s.

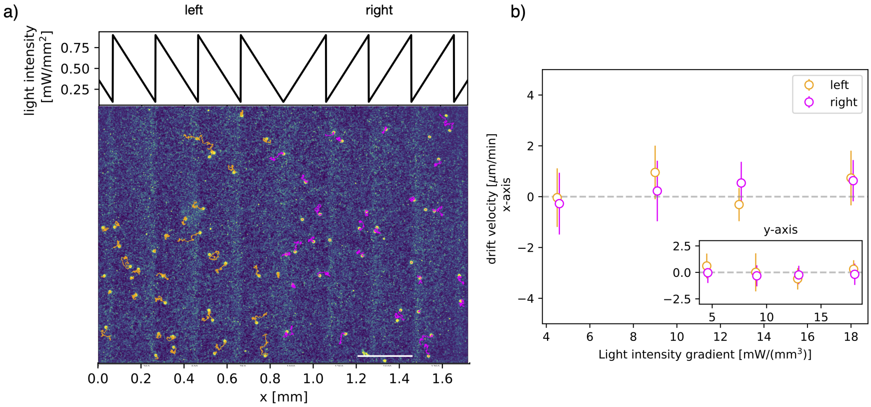

Supplementary Figure 2

Smooth activity gradients do not generate any significant colloidal transport. (a) Trajectories of 10μm-diameter particles under smooth bacterial speed gradients superimposed with a dark field image of the sample. The gradients are along the x-axis and are generated by spatially projecting light with a saw-tooth intensity profile, see the top panel. We projected a negative gradient on the left side of the field of view and a positive one on the right. The particle trajectories are followed for 20 minutes and indicated in orange and magenta for particles starting from the left and right sides, respectively. (b) The particles subjected to different smooth activity gradients do not show any significant drift speed. We report the drift speeds along the x-axis (main panel) and the y-axis (insert) for different activity gradients. Each data is an average of more than 20 particles, and the error bars are the standard errors of the mean.

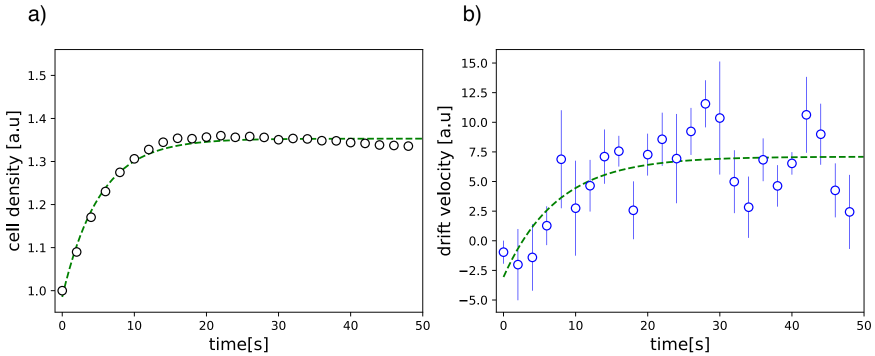

Supplementary Figure 3

Establishment of bacteria density and drift speed during light pattern projection. (a) Cell density in the dark region (I_-) during the first 50s of light pattern projection and starting from a homogeneous concentration. The density is normalized by its initial value. (b) The corresponding drift speed of the particle in the first 50s. The particles start to move as soon as the density in the darker region increases. When the density reaches its maximum, the particle velocity seems also to fluctuate around a constant value. By fitting the two curves with an exponential ($y = A(1 - e^{t/\tau}) + C$), we found a characteristic time τ for the density and velocity establishment of 5 and 7 seconds, respectively. The fits are shown as green dashed lines. After reaching a maximum, the density slowly decreases due to the overall decrease of bacteria from the field of view (caused by the background illumination). Flipping the direction of colloidal transport would require restoring a homogeneous bacteria density around the particle, then flipping the pattern direction and waiting for bacteria pattern formation. Since restoring a homogeneous density around the particles takes only a few seconds, the time of flipping direction is mainly ruled by the transient time τ. Each data point is an average of more than 50 particles, error bars are the standard errors of the mean.

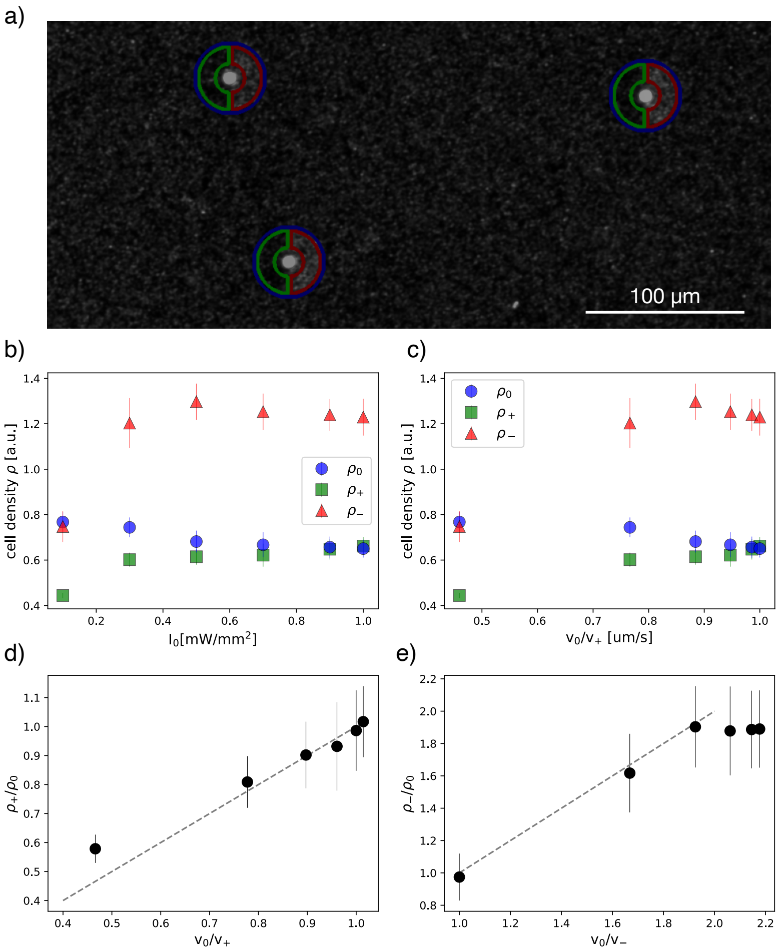

Supplementary Figure 4

Cell density saturates in the dark region while varying bacterial speed in the background. (a) Cell density in the dark, bright, and background regions is measured from the pixel intensities of the dark field images, indicated as red, green, and blue areas, respectively. (b,c) We report the average cell densities in the three regions as a function of the green light intensity I_0 (b) and the velocity of the bacteria v_0 on the background (c) while keeping fixed $v_+ \approx 19$µm/s and $v_- \approx 7$µm/s. Significantly, the density in the dark region ρ_- increases when the velocity v_0 grows but reaches a plateau when $v_0/v_- > 2$ (or $v_0/v_+ > 0.7$). (d)-(e) For an ideal active gas, when the swimming speed varies in space, the stationary density satisfies $\rho v = \text{const}$ [7–9]. This relation seems valid for the bright and background region, where $\rho_+/\rho_0 \approx v_0/v_+$ (d); however, it breaks for the dark region ρ_- (e). The ideal active gas approximation is invalid when the dark region's density exceeds a threshold density and ρ_-/ρ_0 becomes nearly constant. Each data is an average of more than 50 particles, and error bars are the standard errors of the mean.

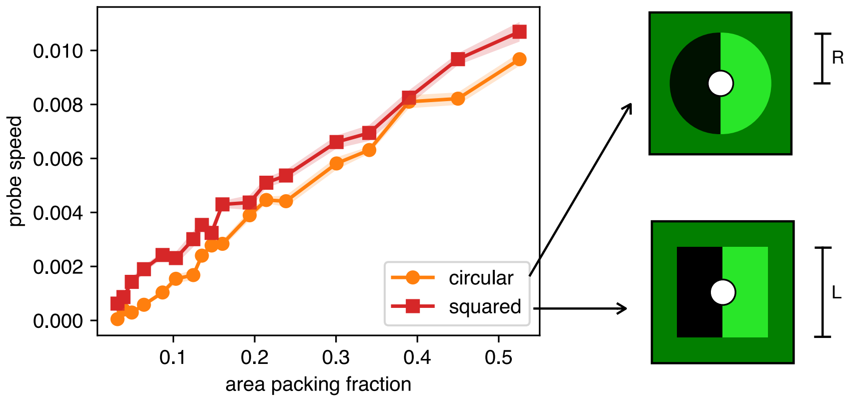

Supplementary Figure 5

Colloidal transport does not depend on the specific shape of the light pattern. We report the drift velocity in simulations when the light pattern has a square or circular shape as a function of cell packing fraction area. The length of the square pattern is $L = R\sqrt{\pi}$, where R is the radius of the circular pattern. In this way, the area of the two shapes is the same. The radius of the circular pattern is R=8a. The data are reported in units of bacteria speed. Each data is time-average over a single trajectory ($N_t = 10^6$ steps with $\Delta t = 10^{-3}$) and then over 20 runs.

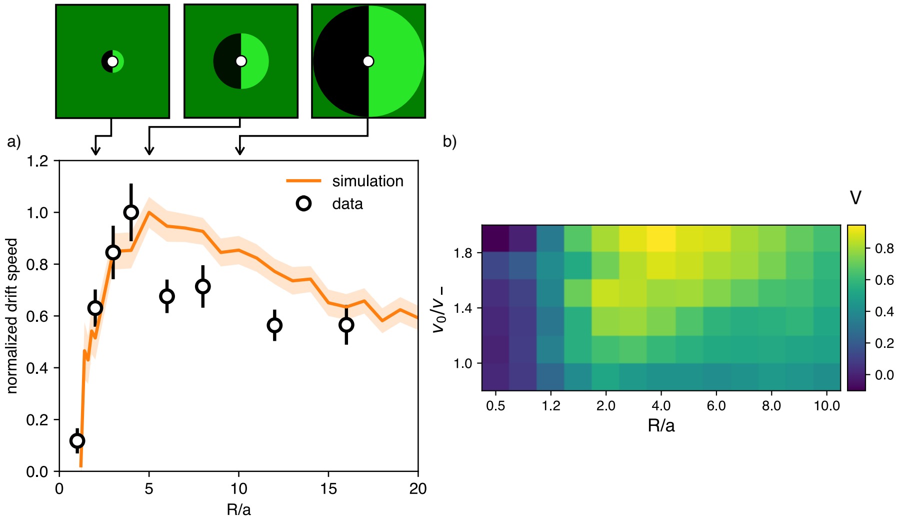

Supplementary Figure 6

Drift speed dependence on modulation disk radius. (a) The drift speed of the particle has a peak when the radius of the light pattern is around four times the particle's radius. Simulations nicely match the experimental trend (orange line-$\phi = 0.44$). We show the drift speed normalized with the maximum value reached in each experiment, $v_{max} = 10.7 \pm 0.6$µm/min. We used for the light pattern $I_- = 0.1$mW/mm^2, $I_0 = 0.7$mW/mm^2, $I_+ = 0.9$mW/mm^2. For the simulations $v_0 = v_p = 2v_-$. Each experimental point is an average over more than 50 particles. Each graph represent the data collected from different samples. For the simulations, we performed a time-average over a single trajectory ($N_t = 10^6$ steps with $\Delta t = 10^{-3}$) and then over $N_s = 10$. Errorbars reported as the shadowed area around the mean. Error bars are the standard errore of the mean for both experiments and simulations. (b) A map showing the normalized drift velocity V as a function of pattern radius R/a and the ratio v_0/v_-, the velocity of bacteria in the background divided by the velocity of bacteria in the dark side. For these simulations we fixed $v_+ = v_0$. The radius R that maximizes the drift velocity increases slightly with v_0/v_-, reaching $R/a \approx 4$ when $v_0/v_+ = 2$ (as for data reported in (a)). All the data reported are calculated performing a time-average over a single trajectory ($N_t = 10^6$ steps with $\Delta t = 10^{-3}$) and then over $N_s = 5$.

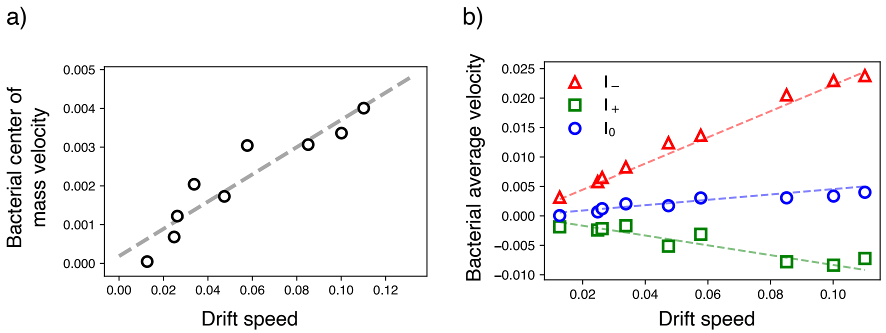

Supplementary Figure 7

Bacterial center of mass speed is proportional to the probe's drift. (a) In simulations, the velocity of bacterial center of mass increases linearly with the drift speed. The experimental data are reported as black dots, while the best linear fit is reported in orange ($\alpha = 0.35$). (b) Average velocity of bacteria in the three regions of the light pattern. We use data coming from simulations where the position of the colloidal particle is shifted with respect to the light pattern position (see main text Fig 3). The data are reported in units of bacteria speed. Each data is time-average over a single trajectory ($N_t = 10^6$ steps with $\Delta t = 10^{-3}$) and then over 10 runs.

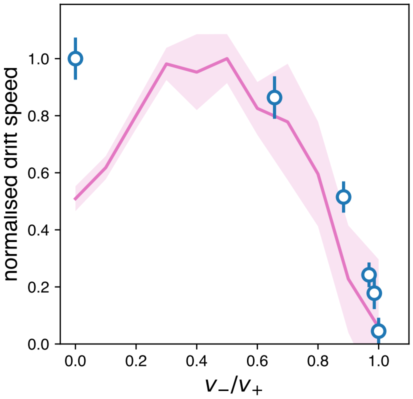

Supplementary Figure 8

Drift speed dependence on swimming speed unbalance. Tracers propulsion dramatically drops when we change v_-, the velocity of bacteria in the darker area of the circular pattern. Exp: blue dots; Sim: pink line. We show the drift speed normalised with respect to the maximum value reached v_{max} = 16µm/min. In the experiments, the bacteria speed was tuned by changing the light intensity. The speed of bacteria were extracted from the speed-intensity curve fitted with the function in SI Fig1). The bacteria speed on the background and bright side was fixed to $v_0 \approx$ 18µm/s ($I_0 = 0.7$mW/mm^2), $v_+ \approx$ 19µm/s ($I_+ = 0.9$mW/mm^2). Each experimental point is an average over more than 50 particles. For the simulations, we perform a time-average over a single trajectory ($N_t = 10^6$ steps with $\Delta t = 10^{-3}$) and then over $N_s = 20$ independent runs. For simulations, errorbars are reported as the shadowed area around the mean. For both experiments and simulations, the error bars are the standard errors of the mean.

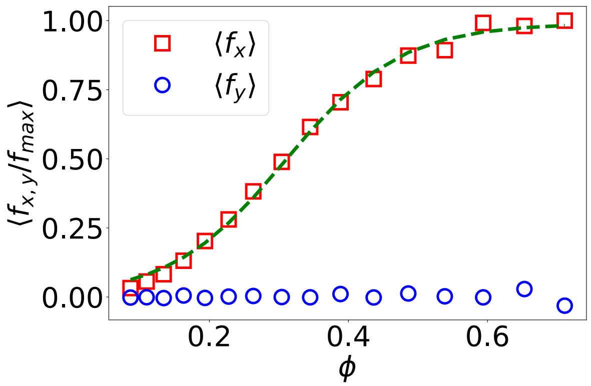

Supplementary Figure 9

Force on a static particle. In numerical simulations, we can keep the particle stationary and measure the force applied by the activity gradient. In red we show the x component and in blue the y component of the force both normalised to the maximum value of f_x and as a function of the bacterial packing fraction ϕ. We found that, even when the particle is fixed, the force applied by the activity gradient is directed towards the region of maximum activity, as in the case of the free particle. The curves are obtained by time averaging individual trajectories ($N_t = 10^6$ steps and $\Delta t = 10^{-3}$) and then averaging over $N_s = 10$ independent runs. The dashed green curve is a fit to a sigmoid function to guide the eye.

References

[1] Angelani, L., Di Leonardo, R., Ruocco, G.: Self-starting micromotors in a bacterial bath. Phys. Rev. Lett. **102**, 048104 (2009). https://doi.org/10.1103/PhysRevLett.102.048104

[2] Paoluzzi, M., Di Leonardo, R., Angelani, L.: Self-sustained density oscillations of swimming bacteria confined in microchambers. Phys. Rev. Lett. **115**, 188303 (2015). https://doi.org/10.1103/PhysRevLett.115.188303

[3] Angelani, L., Maggi, C., Bernardini, M., Rizzo, A., Di Leonardo, R.: Effective interactions between colloidal particles suspended in a bath of swimming cells. Physical review letters **107**(13), 138302 (2011)

[4] Berke, A.P., Turner, L., Berg, H.C., Lauga, E.: Hydrodynamic attraction of swimming microorganisms by surfaces. Physical Review Letters **101**(3), 038102 (2008)

[5] Schwarz-Linek, J., Arlt, J., Jepson, A., Dawson, A., Vissers, T., Miroli, D., Pilizota, T., Martinez, V.A., Poon, W.C.: Escherichia coli as a model active colloid: A practical introduction. Colloids and Surfaces B: Biointerfaces **137**, 2–16 (2016)

[6] Wilson, L.G., Martinez, V.A., Schwarz-Linek, J., Tailleur, J., Bryant, G., Pusey, P., Poon, W.C.: Differential dynamic microscopy of bacterial motility. Physical review letters **106**(1), 018101 (2011)

[7] Frangipane, G., Dell'Arciprete, D., Petracchini, S., Maggi, C., Saglimbeni, F., Bianchi, S., Vizsnyiczai, G., Bernardini, M.L., Di Leonardo, R.: Dynamic density shaping of photokinetic e. coli. Elife **7**, 36608 (2018)

[8] Schnitzer, M.J.: Theory of continuum random walks and application to chemotaxis. Physical Review E **48**(4), 2553 (1993)

[9] Arlt, J., Martinez, V.A., Dawson, A., Pilizota, T., Poon, W.C.: Dynamics-dependent density distribution in active suspensions. Nature communications **10**(1), 1–7 (2019)
