# Supplementary material for: Colloidal transport by light induced gradients of active pressure
Source: Nat Commun. 2023 Jul 13;14:4191. doi: 10.1038/s41467-023-39974-5 (PMC10344923; doi:10.1038/s41467-023-39974-5)
Supplement: Supplementary file 3 — Description of Additional Supplementary Files [file 41467_2023_39974_MOESM3_ESM.pdf]

Description of Additional Supplementary Files

Supplementary Movie 1: Dark-field microscopy video showing several colloidal beads in a bath of light-powered bacteria. When the light pattern is projected, the bacteria accumulate on the slow side (left) and the colloidal beads systematically drift in the direction pointing towards the fast side (right).

Supplementary Movie 2: Video of the 2D bacterial dynamics simulation reproducing the experimental design. A passive particle always lies at the interface between two regions where bacteria have different speeds, while outside of the modulation disk bacteria move with a uniform speed. Blue to pink colormap encodes bacteria orientation. When the bead is centered on the interface a maximum drift is observed.

Supplementary Movie 3: Video of a 2D bacterial dynamics simulation where the bead is fully immersed in the fast half disk and no net drift is observed.

Supplementary Movie 4: Video of a 2D bacterial dynamics simulation where the bead is fully immersed in the slow half disk and no net drift is observed.
